# Evaluation of the impact of a mobile health system on adherence to antenatal and postnatal care and prevention of mother-to-child transmission of HIV programs in Kenya

**DOI:** 10.1186/s12889-015-1358-5

**Published:** 2015-02-07

**Authors:** Ivy Mushamiri, Chibulu Luo, Casey Iiams-Hauser, Yanis Ben Amor

**Affiliations:** One Million Community Health Workers Campaign, the Earth Institute, Columbia University, New York, NY USA; The World Bank, Climate Change Adaptation, Washington, DC USA; Department of Global Health, University of Washington, Seattle, WA USA; The Earth Institute, Columbia University, 475 Riverside Drive, Suite 520, New York, NY 100115 USA

**Keywords:** PMTCT, ANC, mHealth, CHWs, Kenya, HIV

## Abstract

**Background:**

The Millennium Villages Project (MVP) implemented in Western Kenya a mobile Health tool that uses text messages to coordinate Community Health Worker (CHW) activities around antenatal care (ANC) and Prevention of Mother-to-Child Transmission of HIV (PMTCT), named the ANC/PMTCT Adherence System (APAS).

**Methods:**

End-user changes in health-seeking behavior in ANC and postnatal care (PNC) were investigated following registration of 800 women into APAS. These investigations employed interviews of pregnant women or new mothers (n = 67) and CHWs (n = 20). Ordinal logistic regressions and exact binomial tests were used in the routine data analyses (n = 650, health registers).

**Results:**

All CHWs interviewed agreed that APAS helped them track pregnant woman efficiently, compared to paper-based tracking forms. Women registered in APAS reported that CHWs reminded them of appointments more regularly than before its inception.

The routine data analysis showed that among women who had their 1^st^ ANC visit in the 2^nd^ trimester, women who resided in the MVP cluster and were in APAS had:

- 3 times the odds of going for more ANC visits compared to women who were not registered (but resided in the cluster), after adjusting for the mother’s HIV status in the multivariate model (Adjusted OR = 2.58, 95% CI [1.10-6.01]);
- twice the odds of going for more ANC visits compared to women who were not registered and resided outside the cluster (Adjusted OR = 2.37, 95% CI [0.99-5.67])

Among women not registered, residence inside or outside the cluster did not affect the number of ANC visits made (Adjusted OR = 0.86, 95% CI [0.45-1.69]).

The APAS also greatly increased the likelihood of women making the 6 recommended post-delivery baby follow-ups.

For women registered in APAS, the MTCT rate at 18 months was significantly different from that of women not registered, and from the global rate of 30%. Women not registered had a 9% MTCT rate at 18 months regardless of residence, while women registered had a 0% transmission rate at both 9 and 18 months.

**Conclusions:**

The incorporation of mHealth tools in CHW programs can improve adherence to ANC and PNC and enhance PMTCT efforts.

**Electronic supplementary material:**

The online version of this article (doi:10.1186/s12889-015-1358-5) contains supplementary material, which is available to authorized users.

## Background

Despite its virtual elimination in the developed world, vertical transmission of HIV remains a significant challenge in resource-limited settings today. According to WHO estimates, 50% of HIV-positive pregnant women worldwide did not receive prevention of mother-to-child transmission (PMTCT) services in 2010, which resulted in 1000 new pediatric infections daily [1]. The risk of MTCT was shown to be 15-30% among non-breastfeeding populations, and 20-45% among breastfeeding populations in the absence of any PMTCT intervention [2,3]. As a result, the number of children living with HIV currently exceeds 3 million, most of them living in Sub-Saharan Africa [1]. This alarming figure represents 90% of all HIV-infected children globally, with less than a tenth of them being reached with basic health services [4]. Women in low-resource settings are also disproportionately affected, further intensifying the severity of vertical transmission, with fewer health services reaching women in rural areas [5]. The estimated antiretroviral therapy (ART) coverage for PMTCT was only 53% in Kenya for 2012 [6]. Few HIV-exposed infants are being placed on antiretroviral prophylaxis due to high loss to follow-up of women who do not return to the health facilities for antenatal care (ANC) or following birth. ART coverage among eligible children was estimated at only 38% in 2012 for those aged 14 and under, compared to 80.7% for Kenyans aged 15+ years [6]. Cultural and societal factors may influence utilization, including: HIV-related stigmas, fears of status disclosure, lack of financial resources and motivation to access PMTCT services, and poor education or knowledge about PMTCT [7,8].

A health systems approach is essential to heed the call for the virtual elimination of mother-to-child transmission of HIV and reduction of AIDS-related maternal mortality by half by 2015 [9-13]. Integrating PMTCT efforts with community-based approaches can be highly effective at ensuring acceptance and uptake of healthcare services. Additionally, there is an increasing interest in exploring the use of mobile phones and mobile technology to enhance PMTCT efforts [14-16]. The Millennium Villages Project (MVP www.millenniumvillages.org) in Sauri, Western Kenya, mainstreams this integration in PMTCT programs by working directly with Community Health Workers (CHWs). The CHWs follow pregnant women during the entire “PMTCT cascade” from the 1^st^ ANC appointment through to the end of breastfeeding. However, despite the rigorous implementation of a well-defined PMTCT curriculum, CHW activities in Sauri were hampered by a number of challenges. At the inception of MVP in the Kenyan site in 2005, CHWs were unable to follow-up adequately with every woman in the households they cover. Pregnant women were tracked using paper-based methods of data recording, the national norm in Kenya, which proved to be laborious and prone to error. CHWs would often either forget to remind women of their upcoming ANC appointments or not be aware of the next appointment.

The ANC/PMTCT Adherence System (APAS, informally referred to as “PMTCT Module”), which is a mobile Health (mHealth) tool that uses text messages (SMS) to facilitate and coordinate CHW activities around ANC and PMTCT, was implemented in the MVP cluster in October 2010 to help alleviate some of the issues linked with reduced performances. Using any standard mobile phone, readily available in Sub-Saharan Africa [17], CHWs are able to use SMS to register patients during ANC and report their health status to a central system that provides a real-time view of the health of a community. Powerful messaging features help facilitate communication between the members of the health system and an automated alert system helps reduce gaps in treatment. The APAS works by enabling nurses at participating MVP clinics to schedule the next appointment of pregnant woman or their children, and reminders are generated by the system and sent to the corresponding CHW to visit the household and prompt the patient of their upcoming appointment. It also allows for adequate follow-up of those who have defaulted from care.

Since the introduction of the APAS in Sauri in late 2010, over 800 pregnant women have been registered into the system over a period of 2 years and have been followed by CHWs. This study aims to provide a detailed analysis of the impact of the system on retention in the PMTCT cascade and corresponding MTCT rates, and the end-user perceptions of the module by CHWs and pregnant mothers. Since we did not want to single out HIV positive women through our system and since the PMTCT cascade includes ANC visits and baby follow-ups, these outcomes were studied in addition to vertical HIV transmission rates. Overall, we aimed to show that a CHW-centered mHealth technology reminder system can improve PMTCT efforts by increasing uptake of health services, decreasing loss to follow up and decreasing vertical HIV transmission rates in Western Kenya. This study also provides recommendations on how the APAS can be further improved to satisfy the needs of both pregnant women and CHWs.

## Methods

### Study design

#### The APAS software

##### a) The ChildCount + platform

The first step in the study design was the selection of an appropriate platform for the APAS software. The APAS is a component of the ChildCount + (CC+) platform, which is based on the RapidSMS framework, utilizing the Django web framework and programmed in the Python language [18]. The CC+ framework allows for registration of people with unique identifiers (IDs) and generates a persistent database of household members and CHWs [19]. This allows for rapid longitudinal tracking of both clients and their assigned CHWs over time.

The platform requires a laptop server and an SMS modem to run the CC+ framework (both of which were already in place as part of the existing MVP setup), as well as a SMS line, which was negotiated with a local telecom service provider to be toll-free for MVP [20]. All CHWs had been issued mobile phones and SIM cards at the inception of the MVP. No additional materials were necessary for this particular project, and no financial or in-kind incentives were provided to any system user as part of this program.

##### b) APAS workflow^a^

**Figure 1:**
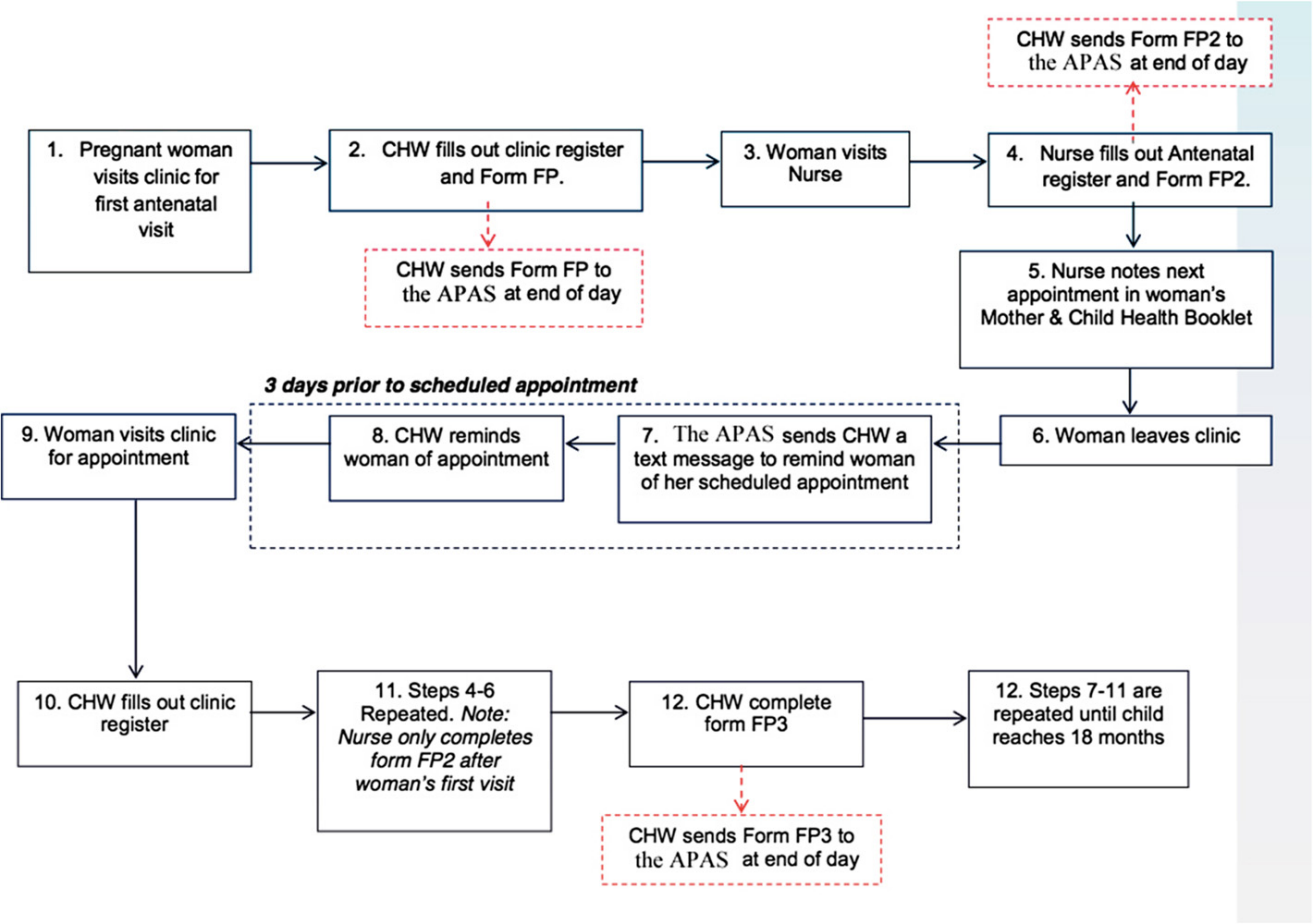
**APAS workflow.** A cascade systematically tracking each pregnant woman presenting herself to the health facility from her 1^st^ ANC visit to 18 months post-delivery and detecting HIV-exposed infants in the process. Forms FP, FP2 and FP3 are attached as appendices (please see Additional files 1, 2 and 3).

Once a platform was selected, the functionalities of the APAS system were defined. Based on MVP standard procedures and in accordance with the Kenyan Ministry of Health, CHWs visit women in their homes on a monthly basis for a variety of household-based interventions. Women who are not already registered in CC+ have their personal information recorded into the system. They are provided with a personalized ID card indicating the health ID numbers of those in their household. Newly pregnant women are referred by the CHW to the local health center for their 1^st^ ANC appointment. Every woman registered in the APAS underwent the same workflow, described diagrammatically in Figure 1.

##### c) APAS software functionalities

During the 1^st^ ANC visit, the CHW first fills out a clinic register and Form FP, which is sent to the APAS at the end of the day (see Additional file 1). The pregnant woman is then directed to the nurse, who fills out the antenatal register and form FP2, which is also sent out to the APAS at the end of the day (see Additional file 2). The antenatal form includes the expected date of delivery, HIV status, and the date of next appointment. Three weekdays before the next scheduled ANC appointment, the system sends an SMS reading **“*Please remind < NAME > to attend their appointment at the health center on < DATE>*”** to the CHW assigned to the expectant mother. The message is identical regardless of the HIV status of the pregnant woman. The expectant mother is then visited in her home by the CHW and reminded of her upcoming appointment. Once the patient is reminded, the CHW sends a confirmation SMS to the APAS system.

If the pregnant woman returns for her appointment as scheduled, a new appointment is created in the system. This marks the previous appointment as completed. Should she fail to visit the clinic, 2 weekdays following her missed appointment, the CHW is sent a 2^nd^ SMS which reads: ***“<NAME > did not attend her appointment at the health center on < DATE > please follow and advise them to return to the health center as soon as possible.”*** The CHW then returns to the pregnant woman to issue a further reminder and confirms his or her visit to the household again via SMS. If another 2 weekdays have passed and the woman has still not attended her ANC visit, the system flags her as a defaulter and she is marked for follow-up. If and when the client eventually returns to the clinic, the defaulter flag is removed from her APAS record.

For each subsequent ANC visit, a new appointment is texted into the APAS structure by the clinic nurse and the reminder/defaulter cycle continues. A follow up visit log (form FP3) is completed by the CHW for every follow-up visit for each pregnant woman in his or her household catchment area (see Additional file 3). A final reminder is sent to the CHW 2 weeks before the expected date of delivery, to review the birth plan with the mother and to provide counseling.

Upon registration of any new birth, the APAS is programmed to check whether the mother is HIV positive. If so, a hidden “HIV-exposed” status is added to the baby’s record. Any outstanding ANC appointment for the pregnant woman is automatically cancelled and replaced with an “under-5” appointment for the child, scheduled at 6 weeks after the date of birth, regardless of HIV status of the mother, to also correspond with the baby’s vaccination schedule. A process of reminders similar to ANC visits is then initiated and continued until the system automatically discontinues the reminder process when the child reaches 18 months of age and graduates out of the PMTCT program.

#### Refining the workflow

A stakeholder analysis was conducted at each participating health facility to refine the PMTCT workflow. These stakeholders included CHWs, Maternal and Child Health (MCH) nurses, pregnant women attending ANC and mothers of children younger than 18 months. The stakeholders were shown the proposed system and workflow and were invited to review and suggest revisions to the final software.

#### Training and rollout

In-service trainings with all 109 CHWs working for MVP at that time were held at each health facility on all aspects of the system, followed by a separate training specifically targeting the 16 clinic-based health providers (midwives and MCH nurses). Trainings particularly emphasized how to fill the required APAS forms. Test SMS were sent to the system to ensure proper formatting, and that the various possible feedbacks from the system were understood. All CHWs were systematically selected and trained on the use of the APAS as part of their regular duties with MVP. Refresher trainings were conducted for all CHWs and clinic-based staff 8 months later, with spot checks conducted by the local program coordinator throughout duration of the program.

### End-user perceptions

Investigations into the end-user perceptions were conducted one year following the implementation of the APAS in Kenya. The main objective of this process was to analyze the effectiveness of the APAS in strengthening the MCH services in the MVP cluster, by increasing the uptake of health services and reducing the vertical HIV transmission rates. The end-user perceptions research process sought to investigate the following question:

*“Was the APAS effective at both simplifying CHW activities and enhancing MCH appointment attendance rates in the MVP cluster?”*

#### Interview process

The appropriate methodology for investigation was to conduct structured one-on-one and focus group interviews with women and CHWs, selected at random across the MVP cluster. Interviews were conducted over a period of 4 weeks, reaching a total of 87 participants (67 women and 20 CHWs^b^).

Data was analyzed by identifying trends in participant responses. Relevant background information was also collected, such as (for women): marital status and total number of pregnancies. Individual interviews were conducted with women in their homes or during their regular clinic visit, and sessions lasted approximately 45 minutes. Three separate focus groups were also arranged, averaging 90 minutes. With CHWs, four separate focus groups were held, each session lasting approximately 2 hours.

#### Set-up

Interviews were set-up using voice recorders, and statements were later transcribed. In cases where individuals did not speak English, a translator was present. Informed consent and participant confidentiality was discussed at the beginning of each interview. It was not assumed that all women selected would be registered in the APAS at the time of interview.

### Routine data analysis

#### Study population

A routine data analysis was conducted to validate results from the investigation of end-user perceptions from interviews. The study population consisted of 650 pregnant HIV-negative and HIV-positive women randomly selected in a stratified sampling manner from health registers in 9 health facilities in the Sauri MVP village cluster. Women from the 10^th^ health facility (Onding Dispensary) were not included because they did not meet the eligibility criteria for the study. Since just over 200 women registered in the APAS met this criteria and funding for the study only allowed sampling similar numbers in other intervention groups, only 650 women were studied. The health registers used included standard registers approved by the Kenyan Ministry of Health: the MCH register, the ANC register, and the HIV Exposed Infant (HEI) register. An electronic record of women registered in the APAS was also used to distinguish women who were enrolled in the system from those who were not. A total of 75 women were sampled from each of 8 health facilities and 50 from 1 facility (Lihanda Health Center). Of the 75 women from each of the 8 facilities, 3 groups were devised as follows: 25 women from outside the MVP cluster (but utilizing health services at MVP health facilities) were not registered in the APAS and were classified “**group 0**”, 25 resided inside the MVP cluster but were not registered in the APAS (**group 1**), while the last 25 both resided in the MVP cluster and were registered in the APAS (**group 2**). The difference between group 0 and group 1 lies not in the quality of clinical services received by the two groups, but in the distance to access those services, as members of group 1 live closer to clinics than members of group 0. Members of group 1 also benefit from a package of community-based healthcare services provided by MVP-employed CHWs, while members of group 0 are either not serviced by CHWs or may have a less systematic and less comprehensive package of community-based healthcare services provided by the Kenyan government or other Non-Governmental Organizations. Of the 50 from Lihanda Health Center, 25 resided inside the MVP cluster but were not registered in the APAS (group 1), while 25 both resided in the MVP cluster and were registered in the APAS (group 2). Lihanda Health Center did not have women meeting eligibility criteria for the study and also satisfying “group 0” requirements. The data was collected between September 2012 and January 2013.

##### ANC visits

**Figure 2:**
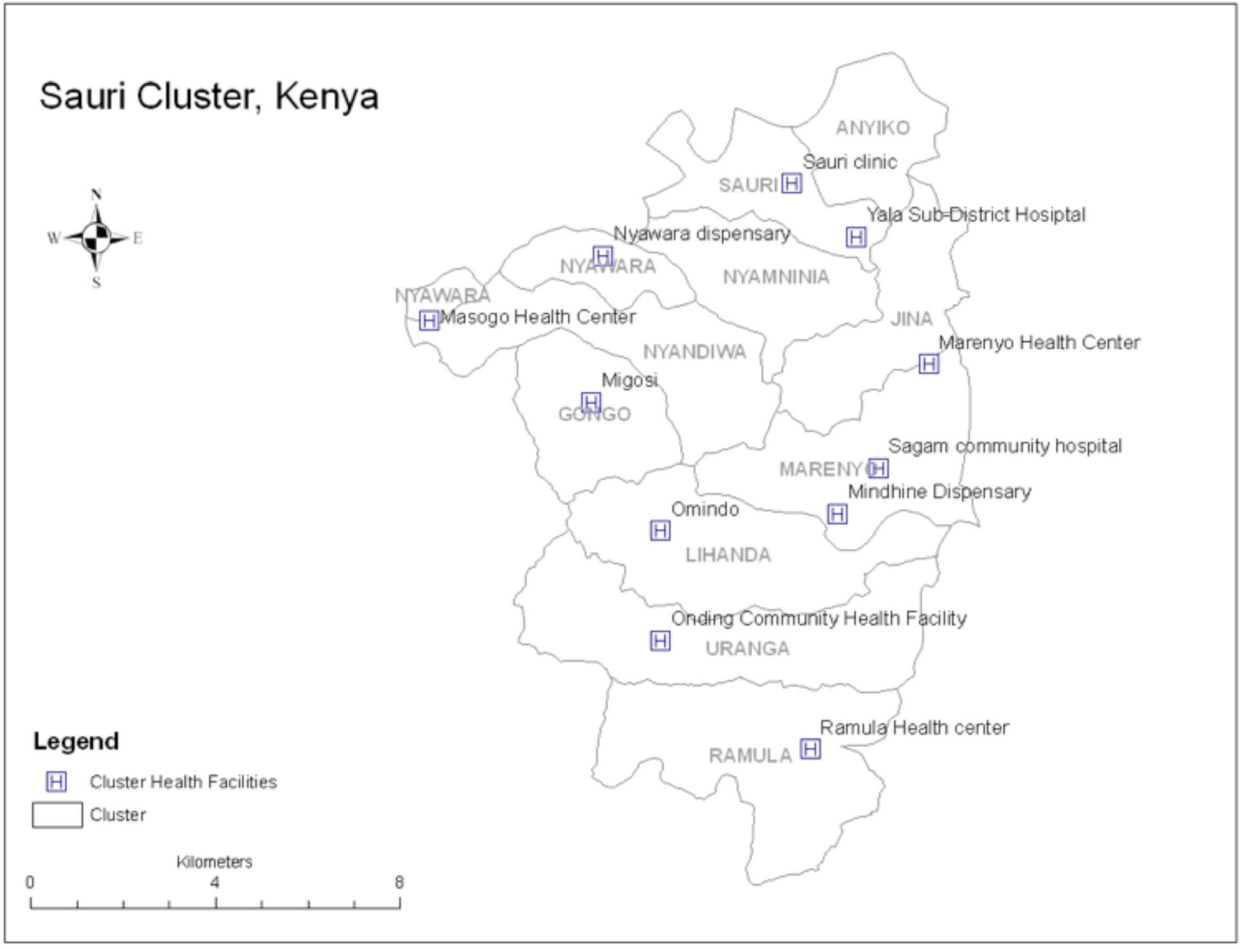
**Map of the Sauri Cluster.** The MVP site in Kenya is divided into 12 sub-locations including Sauri, and is dubbed “Sauri Cluster” because Sauri was where the inception of the package of MVP comprehensive development interventions was first implemented. Each sub-location has its own network of CHWs and the Cluster has 10 health facilities in total, including a sub-district referral hospital.

We conducted a retrospective cohort analysis using data from health registers in 9 health facilities in the Sauri MVP village cluster in the former Nyanza District, Western Kenya [Figure 2]. The link between ANC visits and enrollment in the APAS was investigated. Because enrollment in the APAS can only take place after the women make their 1^st^ ANC visit to the health facility, the total number of ANC visits each woman makes is dependent on how late she is in her pregnancy at the time of her 1^st^ ANC visit. Characteristics of the study population against number of ANC visits were therefore studied according to the trimester of pregnancy at the 1^st^ ANC visit for all the women.

##### Baby follow-ups

The same study population, a total sample of 650 women aged 14–48, was investigated for baby follow-ups for the duration of our study.

#### Inclusion criteria

Only women who had begun ANC care between October 2010 and April 2011 (corresponding to the end of the contract with the telecom provider) were considered for this study. Women enrolled in this period were then followed until 18 months after the baby’s birth. The study only included women with complete information on number of baby follow-ups in order to be able to accurately assess the total number of baby follow-ups each woman made and trace the impact of the intervention all the way to the time the babies were 18 months old for HIV positive women. An attempt was made to randomly sample a total of 30 HIV positive women (10 in each of the 3 intervention groups mentioned above) before sampling HIV negative women at each facility, in order to gather a sample of HIV positive women that closely follows the proportion of HIV positive women in the general population and large enough to study vertical HIV transmission rates. In health facilities where there were less than 10 HIV positive pregnant women reported for the duration of our study in each group, an attempt was made to sample all the HIV positive women, and the deficit (from the intended number of 10) was filled by randomly sampling HIV negative women to bring the total of both HIV positive and negative women to 25 per group, per facility. Women in intervention group 2 were sampled by comparing names (and other identifiers such as village of residence) from the electronic record of women registered in the APAS against the names in the health registers.

#### Outcomes

The outcomes of interest for all women were: number of ANC visits, number of baby follow-ups, baby’s HIV status at 18 months and HIV transmission rates (wherever applicable). Number of ANC visits was measured by adding up all the ANC visits made by the women as recorded in the ANC register. Number of baby follow-ups was measured by adding up all the baby follow-ups recorded in the MCH register. Baby’s HIV status was derived directly from the HEI register. Each baby’s HIV test was done by a PCR test at 6 weeks and 9 months and by an antibody test at 18 months.

#### Exposures

The exposures of interest were 3 different intervention groups described in the study population section.

#### Other covariates

Demographic information was collected from the health registers: age (collected as a continuous variable but split into 7 categories for analysis–according to the different HIV risk-levels per age group), marital status (4 categories), parity [excluding current pregnancy] (collected as a continuous variable but split into 4 categories for analysis) and mother’s HIV status. Other covariates included the facility where the women attended ANC and MCH care, trimester of 1^st^ ANC visit and whether or not there was ART administration for HIV positive women (binary measure).

#### Statistical analysis

Statistical analysis was conducted using SAS 9.3 software. Power was calculated using Power Analysis and Sample Size Software (PASS) version 13. The power calculations were generated using tests for two ordered categorical variables, with Bonferroni adjustment for multiple comparisons (alpha/3). This study was a post-intervention analysis done to evaluate the progress of the APAS intervention that had been introduced in the community, hence a post-hoc power analysis was conducted to study the impact of the intervention. A chi-square analysis or Fisher’s exact test was conducted on all covariates against the outcomes of interest to test for a statistical relationship between any 2 factors. To study the relationship between registration in the APAS and number of ANC visits, an ordinal logistic regression was conducted. This relationship was only studied for women who had their 1^st^ ANC visit in the 2^nd^ trimester of their pregnancy because the total number of ANC visits each woman makes is dependent on how late she is in her pregnancy at the time of her 1^st^ ANC visit, and only 4 women visited the clinic in the 1^st^ trimester. The ordinal levels for number of ANC visits were 1, 2, 3, and 4 or more. To test the relationship between registration in the APAS and number of baby follow-ups, an ordinal logistic regression was conducted. The ordinal levels used for number of baby follow-ups were 0–4, 5 and 6. The 0–4 category was formed by collapsing the sub-categories 0, 1, 2, 3 and 4 because each sub-category had too few women (1, 3, 1, 4 and 24 respectively) to make the proportional odds assumption relevant. For both ordinal logistic regression analyses, a bivariate analysis was conducted first, then other variables were added in a step-wise manner to adjust for potential confounders, and the models with the best fit were presented as the final multivariate models. The vertical HIV transmission rate was calculated by dividing the number of HIV positive babies by the number of HIV positive women in each intervention group and multiplying by 100. To test if the vertical HIV transmission rate for women registered in the APAS was significantly different from that of women not registered in the system and the global rate, an exact binomial test was conducted.

#### Institutional review board

This study was approved by the Columbia University Institutional Review Board (IRB) under protocol number IRB-AAAI4504. The study was also approved by the Ethics Committee at the Kenya Medical Research Institute under protocol number KEMRI/RES/7/3/1 NON-SSC Protocol No 30.

## Results

### Program set up

The duration of set up from concept and design to implementation of the system in the Sauri cluster was 3 months and the pilot was launched in October 2010. The system continued to operate fully until November 2012, and then operated minimally until January 2013. The toll-free SMS service contract was terminated in April 2011, but the disruption in service did not significantly affect the use of the system as most CHWs resorted to using their own phone credit to send text messages to the system and were subsequently reimbursed by the MVP office for this expense.

#### Costs

Investment costs were only $7,000 for the local program manager time. Programmer time, expert technical assistance and all equipment, including cell phones and server were provided by the Earth Institute. Training costs were $100 per training. All SMS to and from the system were provided free of charge to CHWs throughout the pilot. Each cell phone cost $40, the SIM card $2.50 and each SMS cost 1 cent.

### Network coverage in the pilot area

With the exception of only a few households in the community, all of the district homes and clinics were within coverage areas and the network coverage was reliable. Service experienced less than 10% downtime. A major disruption in local network services halted the program for a few weeks in April and May 2011 following the end of the toll-free SMS service contract, which was resolved by reimbursing costs of SMS to CHWs.

### Interviews with CHWs

The MVP site in Kenya is divided into 12 sub-locations [Figure 2], each sub-location with its own network of CHWs. Overall, there were 109 CHWs employed at the MVP site (approximately 9 within each sub-location) at the time of the launch of the pilot. Interviews were conducted 10 months after the start of the project in 4 separate focus group sessions with 20 CHWs i.e. 18% of the total CHW population. Sessions were arranged in the following 3 sub-locations: Sauri (9 CHWs), Nyamninia (4 CHWs) and Gongo (7 CHWs); each session lasting approximately 2 hours. Data collected shows 13 out of 20 participants were female.

Before the APAS was set up, CHWs recorded appointment dates during household visits using paper forms issued by health facilities. CHWs then followed up after the appointment date to check if each pregnant woman attended her appointment. CHWs stated that the paper-based tracking forms were ineffective at helping them remember every woman’s appointment.

After the launch of the APAS, reminders were sent directly to CHWs on their cell phones, which helped CHWs give appointment reminders to women up to 3 days prior to the ANC or follow up visit. All CHWs interviewed agreed that the ability to receive SMS updates on appointments was among the major benefits of the APAS. Based on focus group discussions, CHWs performed on average between 0 and 7 appointment reminders every week.

CHWs were also included in a Closed User Group (CUG), which allowed free phone calls and the ability to send text messages to the system and to each other. Poor battery life of the phones and occasional network failure sometimes hindered CHWs ability to send or receive text messages. CHWs recommended that mothers be integrated into the CUG to help reduce the potential cost of calling outside the CUG, and the provision of solar chargers for the phones.

### Interviews with mothers

A sample size of 67 pregnant women and new mothers^c^ were selected for interviews. Of these, 54 were married and 13 were single. The average number of pregnancies was 3, and the average year of the 1^st^ pregnancy was 2003. Of the 67 women, 38 were interviewed individually and 29 in focus group sessions. Interviews were conducted in the following 7 sub-locations: Anyiko, Gongo, Marenyo, Nyamninia, Nyawara, Ramula and Sauri. Given that the annual estimated number of pregnancies within a sub-location is 4% of a catchment population of 10,000^d^, the women in the sample contributed towards approximately 1.7% of total pregnancies in the site.

Of the participants interviewed individually, 19 out of 38 women were issued yellow ID cards, indicating that approximately 50% of individual interview participants were registered in the APAS at the time of their pregnancy with a unique identifier number. Of the 38 women, 27, including all the 19 women registered in APAS, said that their CHW reminded them of appointments during their regular visit, and therefore they rarely missed their ANC appointments. In addition, all women interviewed were able to verify the next date of their clinic appointment in their MCH booklets, and record dates in their personal diaries or calendars.

In focus group sessions, all participants (29 women) mentioned that they had been issued yellow ID cards, and were registered in the APAS at the time of their pregnancy. Only 4 women reported never receiving appointment reminders from CHWS during their regular visit. Of all participants, 23 women noted that they had a mobile phone, and most were able to send, receive and read text messages. All agreed that they would be willing to opt-in to a service that allowed them to have reminders sent directly to their phones.

The women were also asked to share what messages their CHW communicated to them during their regular visits. Common responses were that CHWs educated them on malaria and HIV prevention methods. Furthermore, 55 out of all 67 women in the sample (82% of participants) said that their CHW visited them regularly i.e. every 1 or 2 months. All women agreed that CHWs were reminding them of clinic appointments more regularly than before the introduction of the APAS. Those who rarely saw their CHW shared that they had not seen any changes.

### Routine data

#### ANC visits

##### Study population

The biggest age category of women in this sample of our study was the 20–24 year olds (34.8% of participants), while the smallest was the less than 15 years old category (0.9% of participants) [See Table 1]. Most participants came from Masogo Dispensary (12.9%), while Lihanda Health Center had the smallest representation as expected, given that it had the smallest sample to begin with (8.6%). The intervention group 0 made up 33.6% of the total participants, while 37.9% were in the intervention group 1, and 28.5% were in the intervention group 2. The HIV prevalence in the investigated population was 27.3%, with 95 being HIV positive and 253 being HIV negative. Most women were married (91.4%) and had 1–3 children prior to the current pregnancy (56.9%).

**Table 1:** **Population characteristics by number of ANC visits: women presenting for 1st ANC in 2nd trimester**

**Characteristic**	**Total**		**≥4**		**<4**		**p-value**
	**N = 348**		**N = 293**		**N = 55**		
	**N**	**%**	**n**	**%**	**n**	**%**	
**Age (Years)**							
<15	3	0.9	3	1.0	0	0.0	0.9555
15-19	73	21.0	61	20.8	12	21.8	
20-24	121	34.8	101	34.5	20	36.4	
25-29	69	19.8	57	19.5	12	21.8	
30-34	53	15.2	46	15.7	7	12.7	
35-39	25	7.2	22	7.5	3	5.5	
40-44	4	1.2	3	1.0	1	1.8	
**Facility**							
Gongo HC	42	12.1	37	12.6	5	9.1	0.0979
Lihanda HC	30	8.6	23	7.9	7	12.7	
Marenyo HC	37	10.6	33	11.3	4	7.3	
Masogo Disp	45	12.9	37	12.6	8	14.6	
Mindhine Disp	38	10.9	36	12.3	2	3.6	
Nyawara HC	39	11.2	35	12.0	4	7.3	
Ramula HC	42	12.1	34	11.6	8	14.6	
Sauri HC	35	10.1	24	8.2	11	20.0	
Yala SDH	40	11.5	34	11.6	6	10.9	
Intervention*							
0	117	33.6	97	33.1	20	36.4	**0.0346**
1	132	37.9	105	35.8	27	49.1	
2	99	28.5	91	31.1	8	14.6	
**Mother's HIV Status**							
Negative	253	72.7	219	74.7	34	61.8	**0.0483**
Positive	95	27.3	74	25.3	21	38.2	
**Marital Status**							
Married	318	91.4	269	91.8	49	89.1	*0.5880*
Widowed	1	0.3	1	0.3	0	0.0	
Single	28	8.1	22	7.5	6	10.9	
Separated	1	0.3	1	0.3	0	0.0	
**Parity**							
0	86	24.7	70	23.9	16	29.1	*0.7284*
1-3	198	56.9	168	57.3	30	54.6	
4-6	59	17.0	51	17.4	8	14.6	
≥7	5	1.4	4	1.4	1	1.8	
**Key**							

*Intervention: 0 = outside cluster and outside *APAS*, 1 = in cluster and outside *APAS*, 2 = in cluster and in *APAS.*

Italicized p-value = Derived from Fisher's exact test (not chi-square).

Bold figures = Significant association.

##### Chi-square and Fisher tests

Table 1 presents results for women who made their 1^st^ ANC visit in the 2^nd^ trimester of their pregnancy (348 of the 650 women). The chi-square or Fisher’s exact test show that there were no statistically significant differences in the distribution of most population-level characteristics between women who made the nationally recommended 4 or more ANC visits and those who made less than 4 ANC visits (p-values >0.05) except for the intervention groups and mother’s HIV status. The p-values for the intervention groups and mother’s HIV status (<0.05) show that there was a statistically significant association between the number of ANC visits made and both the intervention group and the mother’s HIV status. These characteristics are also illustrated graphically in Additional file 4.

##### Ordinal logistic regression analysis

Table 2 summarizes the univariate and multivariate logistic regression analyses of the intervention groups against number of ANC visits. The crude (univariate) ordinal logistic regression analysis showed a statistically significant positive association between intervention 2 compared to 1 and higher number of ANC visits (OR = 2.91, 95% CI [1.26-6.73]), that is, among women who had their 1^st^ ANC visit in the 2^nd^ trimester of their pregnancy and resided in the MVP cluster, women who were in the APAS had 3 times the odds of going for more, rather than less, ANC visits compared to women who were not in the APAS. This relationship remained significant and of similar magnitude even after adjusting for the mother’s HIV status in the multivariate model (Adjusted OR = 2.58, 95% CI [1.10-6.01]). The power of this analysis was 0.91.

**Table 2:** **Ordinal logistic regression: intervention with ANC visits (women presenting for 1st ANC in 2nd trimester)**

	**Crude analysis**		Adjusted analysis**	
	**OR**	**95% CI**	**OR**	**95% CI**
Intervention*				
1 vs 0	0.78	(0.41-1.49)	0.86	(0.45-1.69)
2 vs 0	2.30	(0.97-5.48)	2.37	(0.99-5.67)
2 vs 1	**2.91**	**(1.26-6.73)**	**2.58**	**(1.10-6.01)**
**Key**				

*Intervention: 0 = outside cluster and outside *APAS*, 1 = in cluster and outside *APAS*, 2 = in cluster and in *APAS.*

**Adjusted for mother's HIV status.

Bold figures = Significant association.

There was, however, no statistically significant relationship between intervention 1 compared to 0 in both the univariate and multivariate models (OR = 0.78, 95% CI [0.41-1.49], Adjusted OR = 0.86, 95% CI [0.45-1.69] respectively), meaning residence inside or outside the cluster did not affect the number of ANC visits made (power = 0.05). The relationship between intervention 2 compared to 0 was nearly significant in both the crude and adjusted analysis (OR = 2.30, 95% CI [0.97-5.48], Adjusted OR = 2.37, 95% CI [0.99-5.67] respectively), hence among women who had their 1^st^ ANC visit in the 2^nd^ trimester of their pregnancy, women who were in the APAS and resided in the MVP cluster had twice the odds of going for more, rather than less, ANC visits compared to women who were not in the APAS and resided outside the MVP cluster (power = 0.58).

### Baby follow-ups

#### Study population

The total sample investigated for baby follow-ups consisted of 650 women aged 14–48. The median age was 24 (IQR 20–28). The biggest age category was the 20–24 year olds (34.9% of participants), while the smallest was the 45–49 year olds category (0.2% of participants) [See Table 3]. Lihanda Health Center had the smallest representation of women (50 participants) while the rest of the facilities contributed an equal number of participants (75 each). The intervention group 0 was made up of 200 women, while the intervention groups 1 and 2 each had 225 women. Most women in this sample made their 1^st^ ANC visit in the 2^nd^ trimester of their pregnancy (348 in total). The HIV prevalence in the investigated population was 27.1%, with 176 being HIV positive and 474 being HIV negative. Most women were married (91.2%) and had 1–3 children prior to the current child (60.3%). These characteristics are also illustrated graphically in Additional file 5.

**Table 3:** **Population characteristics by number of baby follow-ups**

**Characteristic**	**Total**		**≥6**		**<6**		**p-value**
	**N = 650**		**N = 556**		**N = 94**		
	**N**	**%**	**n**	**%**	**n**	**%**	
**Age (Years)**							
<15	3	0.5	3	0.5	0	0.0	0.0922
15-19	127	19.5	108	19.4	19	20.2	
20-24	227	34.9	201	36.2	26	27.7	
25-29	148	22.8	123	22.1	25	26.6	
30-34	91	14.0	73	13.1	18	19.2	
35-39	47	7.2	43	7.7	4	4.3	
40-44	6	0.9	5	0.9	1	1.1	
45-49	1	0.2	0	0.0	1	1.1	
**Facility**							
Gongo HC	75	11.5	64	11.5	11	11.7	**<0.0001**
Lihanda HC	50	7.7	46	8.3	4	4.3	
Marenyo HC	75	11.5	72	13.0	3	3.2	
Masogo Disp	75	11.5	58	10.4	17	18.1	
Mindhine Disp	75	11.5	66	11.9	9	9.6	
Nyawara HC	75	11.5	72	13.0	3	3.2	
Ramula HC	75	11.5	71	12.8	4	4.3	
Sauri HC	75	11.5	49	8.8	26	27.7	
Yala SDH	75	11.5	58	10.4	17	18.1	
Intervention*							
0	200	30.8	150	27.0	50	53.2	**<0.0001**
1	225	34.6	189	34.0	36	38.3	
2	225	34.6	217	39.0	8	8.5	
**Trimester of 1st ANC Visit**							
1st	4	0.7	4	0.8	0	0.0	*0.9517*
2nd	348	56.9	297	56.9	51	56.7	
3rd	260	42.5	221	42.3	39	43.3	
*Missing*	38						
**Mother's HIV Status**							
Negative	474	72.9	411	73.9	63	67.0	0.1638
Positive	176	27.1	145	26.1	31	33.0	
**Marital Status**							
Married	593	91.2	504	90.7	89	94.7	*0.5720*
Widowed	2	0.3	2	0.4	0	0.0	
Single	54	8.3	49	8.8	5	5.3	
Separated	1	0.2	1	0.2	0	0.0	
**Parity**							
0	153	23.5	129	23.2	24	25.5	0.4654
1-3	392	60.3	339	61.0	53	56.4	
4-6	97	14.9	80	14.4	17	18.1	
≥7	8	1.2	8	1.4	0	0.0	
**Key**							

*Intervention: 0 = outside cluster and outside *APAS*, 1 = in cluster and outside *APAS*, 2 = in cluster and in *APAS.*

Italicized p-value = Derived from Fisher’s exact test (not chi-square).

Bold figures = Significant association.

#### Chi-square and Fisher tests

Characteristics of the study population against number of baby follow-ups are presented in Table 3. The chi-square or Fisher’s exact test show that there were no statistically significant differences in the distribution of most population-level characteristics between women who made the nationally recommended 6 or more baby follow-ups and those who made less than 6 baby follow-ups (p-values >0.05) except for the facility at which they sought health care and the intervention group. The p-values for the facility at which they sought health care and the intervention group (both < 0.0001) show that there was a strong statistically significant association between the number of baby follow-ups made and the intervention group, and between the number of baby follow-ups made and facility at which the women sought health care.

#### Ordinal logistic regression analysis

Table 4 summarizes the univariate and multivariate logistic regression analyses of the intervention group against number of baby follow-ups. The crude (univariate) ordinal logistic regression analyses showed a statistically significant positive association between higher ordered interventions (1 vs 0, 2 vs 0 and 2 vs 1) and higher number of baby follow-ups (OR = 1.75, 95% CI [1.09-2.82], OR = 9.00, 95% CI [4.15-19.51], OR = 5.14, 95% CI [2.34-11.33] respectively). In other words, women who resided inside the MVP cluster had twice the odds of going for more, rather than less, baby follow-ups compared to women who resided outside the MVP cluster; women who were in the APAS and resided in the MVP cluster had 9 times the odds of going for more, rather than less, baby follow-ups compared to women who were not in the APAS and resided outside the MVP cluster; among women who resided in the MVP cluster, women who were in the APAS had 5 times the odds of going for more, rather than less, baby follow ups compared to women who were not in the APAS.

**Table 4:** **Ordinal logistic regression analysis of intervention with baby follow-ups (all women)**

	**Crude analysis**		Adjusted analysis**		Adjusted analysis***	
	**OR**	**95% CI**	**OR**	**95% CI**	**OR**	**95% CI**
Intervention*						
1 vs 0	**1.75**	**(1.09-2.82)**	**1.84**	**(1.13-2.98)**	**1.87**	**(1.15-3.05)**
2 vs 0	**9.00**	**(4.15-19.51)**	**8.96**	**(4.13-19.41)**	**8.99**	**(4.15-19.49)**
2 vs 1	**5.14**	**(2.34-11.33)**	**4.26**	**(1.91-9.48)**	**4.00**	**(1.79-8.93)**
**Key**						

*Intervention: 0 = outside cluster and outside APAS, 1 = in cluster and outside APAS, 2 = in cluster and in APAS.

**Adjusted for Mother's HIV Status.

***Adjusted for Mother's HIV Status and Parity.

Bold figures = Significant association.

The multivariate models were similar to the univariate models, showing strong statistically significant positive associations between higher ordered interventions (1 vs 0, 2 vs 0 and 2 vs 1) and higher number of baby follow-ups (Adjusted OR = 1.84, 95% CI [1.13-2.98], Adjusted OR = 8.96, 95% CI [4.13-19.41], Adjusted OR = 4.26, 95% CI [1.91-9.48] respectively in the model adjusting for mother’s HIV status), and (Adjusted OR = 1.87, 95% CI [1.15-3.05], Adjusted OR = 8.99, 95% CI [4.15-19.49], Adjusted OR = 4.00, 95% CI [1.79-8.93] respectively in the model adjusting for mother’s HIV status and parity).

### Vertical HIV transmission

#### Study population

Among HIV positive women with data on baby’s HIV status at 18 months, the biggest age category was the 25–29 year olds (33% of participants), while the smallest was the 40–44 year olds category (1.1% of participants) [see Table 5]. Yala Sub-District Hospital had the most participants (29.6%) while Masogo dispensary had the smallest representation of women (1.1%). The intervention group 0 was made up of 33 women; the intervention group 1 had 35 women; and the intervention group 2 had 20 women. Most women in this sample made their 1^st^ ANC visit in the 2^nd^ trimester of their pregnancy (57.1%). Most women made 6 or more baby follow-ups (85.2%), and less than 4 ANC visits (51.4%). Most women were married (96.6%) and had 1–3 children prior to the current child (77.3%). The majority of the HIV positive women received ART administration (69.1%). These characteristics are also illustrated graphically in Additional file 6.

**Table 5:** **Population characteristics by baby's HIV status at 18 months**

**Characteristic**	**Total**		**Positive**		**Negative**		**p-value**
	**N = 176**		**N = 6**		**N = 82**		
	**N**	**%**	**n**	**%**	**n**	**%**	
**Age (Years)**							
15-19	4	4.6	0	0.0	4	4.9	*0.9204*
20-24	23	26.1	1	16.7	22	26.8	
25-29	29	33.0	2	33.3	27	32.9	
30-34	21	23.9	2	33.3	19	23.2	
35-39	10	11.3	1	16.7	9	11.0	
40-44	1	1.1	0	0.0	1	1.2	
*Missing*	88						
**Facility**							
Gongo HC	3	3.4	0	0.0	3	3.7	*0.0914*
Lihanda HC	2	2.3	0	0.0	2	2.4	
Marenyo HC	17	19.3	4	66.7	13	15.9	
Masogo Disp	1	1.1	0	0.0	1	1.2	
Mindhine Disp	3	3.4	0	0.0	3	3.7	
Nyawara HC	19	21.6	0	0.0	19	23.2	
Ramula HC	13	14.8	2	33.3	11	13.4	
Sauri HC	4	4.6	0	0.0	4	4.9	
Yala SDH	26	29.6	0	0.0	26	31.7	
*Missing*	88						
Intervention*							
0	33	37.5	3	50.0	30	36.6	*0.4998*
1	35	39.8	3	50.0	32	39.0	
2	20	22.7	0	0.0	20	24.4	
*Missing*	88						
**Trimester of 1st Visit**							
1st	0	0.0	0	0.0	0	0.0	*0.0677*
2nd	48	57.1	5	100.0	43	54.4	
3rd	36	42.9	0	0.0	36	45.6	
*Missing*	92						
**Baby Follow Up's**							
<6	13	14.8	2	13.3	11	13.4	*0.2144*
≥6	75	85.2	4	66.7	71	86.6	
*Missing*	88						
**ANC Visits**							
<4	45	51.4	0	0.0	45	54.9	**0.0112**
≥4	43	48.9	6	100.0	37	45.1	
*Missing*	88						
**Marital Status**							
Married	85	96.6	6	100.0	79	96.3	*1.0000*
Widowed	1	1.1	0	0.0	1	1.2	
Single	2	2.3	0	0.0	2	76.8	
Separated	0	0.0	0	0.0	0	0.0	
*Missing*	88						
**Parity**							
0	8	9.1	0	0.0	8	9.8	0.1064
1-3	68	77.3	5	83.3	63	76.8	
4-6	11	12.5	0	0.0	11	13.4	
≥7	1	1.1	1	16.7	0	0.0	
*Missing*	88						
**ART Administration**							
No	58	69.1	5	83.3	53	68.0	*0.6608*
Yes	26	31.0	1	16.7	25	32.1	
*Missing*	92						
**Key**							

*Intervention: 0 = outside cluster and outside *APAS*, 1 = in cluster and outside *APAS*, 2 = in cluster and in *APAS.*

Italicized p-value = Derived from Fisher's exact test (not chi-square).

Bold figures = Significant association.

#### Chi-square and Fisher tests

Among the 176 women who were HIV positive, vertical HIV transmission was first studied using a chi-square analysis and Fisher’s exact tests. Characteristics of the study population against the baby’s HIV status at 18 months are presented in Table 5. In this sample of HIV positive women, 88 did not have data on the baby’s HIV status at 18 months. The chi-square or Fisher’s exact tests show that there were no statistically significant differences in the distribution of most population-level characteristics between women who had an HIV positive baby at 18 months and those who had an HIV negative baby at 18 months (p-values >0.05) except for the number of ANC visits (p-value <0.05).

#### Transmission rates

The chi-square analysis and Fisher’s exact tests did not show a statistically significant relationship between the intervention levels and baby’s HIV status at 18 months [see Table 5]. However, an exact binomial test for proportions showed that the vertical HIV transmission rate at 18 months for women registered in the APAS was significantly different from that of women not registered in the APAS and was significantly different from the global vertical HIV transmission rate of 30% (data not shown). The intervention groups 1 and 0 each had 3 HIV positive babies at 18 months (9% transmission rate) while intervention group 2 had a 0% transmission rate [see Table 5], hence none of the HIV positive women who both resided in the MVP cluster and were in the APAS transmitted the HIV virus to their babies. Although 18 months is a more definite time point to investigate vertical HIV transmission as most babies are weaned from breastfeeding, transmission rates were also measured at an earlier time point (when the babies were 9 months) with a bigger sample (only 10 women missing data as opposed to 88), and the transmission rates for intervention groups 0, 1 and 2 were 14.4%, 8.3% and 0% respectively (data not shown).

## Discussion

Mobile telephony has become ubiquitous in Africa and more people have access to mobile phones in Sub-Saharan Africa than to safe drinking water or electricity [21]. Increasingly, development programs are harnessing the potential of mobile phones and mobile technology for health system improvement, including for nutrition, tuberculosis, malaria [22] and maternal and child health [14-16,23-26]. In 2010, the Joint United Nations Programme on HIV/AIDS (UNAIDS) called for the virtual elimination of mother-to-child transmission of HIV and reduction of AIDS-related maternal mortality by half by 2015 [9-13]. Our paper shows that the implementation of the APAS in MVP’s Sauri cluster in Western Kenya (formerly Nyanza Province) has proven effective in tracking and improving the treatment-seeking behavior of pregnant women, during pregnancy and up to 18 months following delivery, and could therefore effectively complement on-going PMTCT efforts to reach the goal of virtual elimination by 2015.

In order to ensure a safe pregnancy, 4 ANC visits are standardly recommended, including in Kenya [27,28]. Additionally, the 1^st^ visit is recommended during the 1^st^ trimester (around 15 weeks) to allow for an appropriate spacing of the ANC visits throughout the pregnancy [27]. In our study however, most women presented during the 2^nd^ trimester, as is the norm in rural sub-Saharan Africa settings [27]. Based on routine based data from the sample of women who had their 1^st^ ANC visit in the 2^nd^ trimester of their pregnancy and resided in the MVP cluster, women who were enrolled in the APAS had 3 times the odds of going for more ANC visits rather than less, compared to women not enrolled in the APAS after adjusting for the mother’s HIV status. Therefore, the strength of our proposed mHealth solution lies in the span of its impact to all women, regardless of their HIV status. As shown by our study, women enrolled in the APAS were more likely to undergo the 4 recommended ANC visits compared to women not enrolled in the APAS. In our sample, residence inside or outside the cluster did not seem to affect the number of ANC visits made, but this could be explained by low statistical power in the analysis comparing groups 1 and 0, and by the fact that women from outside the cluster attend MVP clinics, which all focus strongly on the need for the 4-recommended visits for a safe pregnancy. Also, there are CHWs in other areas around MVP that are part of the national program, but they are not structured in the same way as MVP and their curriculum is different (less complete and does not include PMTCT).

The interesting caveat of our mHealth system is that it can only effectively work if women present to the ANC clinic earlier rather than later. From data collected during interviews and focus groups, women shared that family planning and HIV prevention were among the main educational messages disseminated by CHWs during their regular visits. While this data suggests that women in the community are aware of the importance of fulfilling their clinic appointments, only 4 women (0.6%) in the sample used for routine based data analysis made their 1^st^ ANC appointment in their 1^st^ trimester, underlining a stark difference between knowledge and practice. Additionally, there may be cultural reasons that further explain or justify why pregnant women tend to wait until the 2^nd^ trimester before presenting to a health clinic [8]. The method of counseling that CHWs adopt in the community to convey messages around ANC and PMTCT are as important as the content of the message itself and these methods empower pregnant women to realize the importance of clinic visits for both themselves and their children. CHWs are therefore a critical piece in the system and their role is not just focused on the adherence component, rather is continuous throughout the year as they also allow the mHealth system to be more effective by convincing pregnant women to present to clinics earlier than the 2^nd^ trimester. We believe that, whenever possible, CHWs should complement any mHealth tool designed to increase adherence, whether it be focused on HIV, TB or even chronic diseases such as diabetes.

In order to ensure appropriate and complete postnatal care, 6 baby follow-up visits are recommended, covering all mandatory vaccinations, nutritional assessments and Early Infant Diagnosis for HIV-exposed infants [29]. In our study, we found that women who resided inside the MVP cluster had twice the odds of going for more baby follow-ups rather than less, compared to women who resided outside the MVP cluster. Additionally, among women residing in the MVP cluster, women who were registered in the APAS had 4 times the odds of going for more baby follow-ups rather than less, compared to women not registered in the APAS, after adjusting for mother’s HIV status and parity. The APAS therefore greatly increases the likelihood of women making the recommended 6 baby follow-ups. This could lead to improved infant and young child health outcomes. Interestingly, we also showed that there was a statistically significant association between number of baby follow-ups and the facility at which women sough health services (a similar association was not uncovered for ANC visits). This could be linked to a variety of reasons, ranging from the perceived quality of services provided, the friendliness of the staff or more simply, the wait time during special days organized around infant and young child care (vaccination, nutritional assessment etc.). Additionally, in our sample, residence inside or outside the cluster did not seem to affect the number of ANC visits made but there was a statistical difference when looking at baby follow-ups. This could possibly indicate that distance to the health center does not seem to impact access to ANC, but does have a negative influence on the future follow-ups the mother undertakes with her baby. Any program interested in improving their follow-up rates should therefore also look into the improvement of these important factors.

One of the goals envisioned for the APAS upon its inception was to help alleviate vertical HIV transmission rates while also providing additional functionalities to reach all pregnant women, regardless of HIV status. Our routine based data analysis shows that while the APAS does not disclose to CHWs a woman’s HIV status at any point during her pregnancy, through registration in the system, CHW activities are proving to be particularly effective in reaching HIV positive mothers. While we could not show a statistically significant relationship between enrollment in the APAS and a baby’s HIV status at 18 months using a chi-square analysis or Fisher’s exact test, an exact binomial test for proportions showed that the vertical HIV transmission rate at 18 months for women registered in the APAS was significantly different from that of women not registered in the system as well as significantly different from the global vertical HIV transmission rate of 30%.

All 20 HIV-positive women in the randomly selected sample of registered APAS users living in the MVP cluster with complete data on baby’s HIV status at 18 months gave birth to HIV negative babies, compared to the 9% vertical HIV transmission rate for women not registered in the APAS but living in the cluster, and a similar rate for women outside the cluster, suggesting that the system is alleviating MTCT in the community. Transmission rates at 9 months following birth were 14.4% for women not registered in the APAS and residing outside the MVP cluster, 8.3% for women not registered in the APAS but residing inside the MVP cluster, and 0% for women both registered in the APAS and residing inside the MVP cluster. This suggests that the efforts undertaken by MVP to reduce MTCT within the cluster have effectively close-to halved the vertical HIV transmission rate. However, only through the use of the APAS was it possible to eliminate transmission altogether, in the sample we analyzed. Interestingly, we also showed that the number of ANC visits during pregnancy was directly correlated to the transmission rate, which confirms what was intuitively anticipated.

Our study had several limitations that were independent from our work. PMTCT indicators improve as women attend their 1^st^ ANC appointment during the earliest stages of pregnancy i.e. within the 1^st^ trimester. If a pregnant woman makes her first appointment during later trimesters, she will have fewer opportunities to make the 4 ANC visits required before delivery. As such, our study could only effectively study women who made their 1^st^ ANC visit in the 2^nd^ trimester of their pregnancy (as only 4 women visited the clinic in the 1^st^ trimester). When all women were considered regardless of trimester of 1^st^ ANC visit, the statistical relationship between number of ANC visits made and registration in the APAS was not valid. This could be explained by a reduced opportunity for the health system to adequately provide effective services when the women presented later in their pregnancy. Interestingly, even when women were presenting later, they were still more likely to attend up to 4 ANC appointments during the course of their pregnancy if they were registered in the APAS. Overall, this stresses the importance for nurses and CHWs to continue to counsel women and build awareness around PMTCT to encourage more women to present themselves at the clinic as early as their 1^st^ trimester.

Another concern is that HIV positive women were oversampled in this study in an effort to match the regional HIV prevalence [15.1% in Nyanza Province in 2012] [30] and sample a population large enough to study vertical HIV transmission rates. These efforts, however, resulted in an increased overall HIV prevalence in our sample to 27.3% and possibly led to a bias in our results. We attempted to decrease this bias by adjusting for mother’s HIV status as a confounder in our final models. A separate analysis conducted with only HIV negative women did not significantly change the results (data not shown).

Since this study was a post-intervention analysis, a post-hoc power analysis for the main outcome was conducted. This analysis showed that the power for the relationship between intervention groups 1 and 0 (women not registered in APAS and residing inside and outside the MVP cluster respectively) was very low (0.05). This particular analysis was not powered enough to detect a significant relationship between the 2 groups. However, the study may have overall been over powered since a power of 0.91 was observed in the analysis of the relationship between intervention groups 2 and 1 (women residing in the MVP cluster but registered and not registered in APAS respectively). Since a large effect size (OR of 2.91 in the crude analysis for ANC visits) was observed, however, the findings are still an important indicator of the impact of an mHealth intervention on aiding PMTCT efforts.

Another limitation is that the study only included women with complete information on baby follow-ups in order to be able to accurately assess the total number of baby follow-ups each woman made and trace the impact of the intervention all the way to the time the babies were 18 months old for HIV positive women. This could have introduced a bias if there was something characteristic about women with missing data points on baby follow-ups. The MCH nurses however attribute missing data points on baby follow-ups in the MCH register to merely operational failure. Additionally, since this selection process was applied to all three groups, this possible bias is not likely to impact the difference between groups.

It is also important to note that operationally, CHWs were supposed to register all pregnant women in their sub-location into the APAS, hence “group 1” (women who resided inside the MVP cluster but were not registered in the APAS) was a result of CHWs or clinical staff not adhering to the MVP protocol. The fact that CHWs continued to follow up with this group of women during pregnancy to provide appointment reminders (which they would record in their MCH booklets) and also educate them on HIV and family planning as they did before the introduction of APAS could potentially have biased our results, but this group was different from “group 2” (women who both resided in the MVP cluster and were registered in the APAS) in the fact that group 2 received more systematic and more frequent reminders as demonstrated in Figure 1.

The MVP team also encountered several problems with the system, mainly due to the suspension of the agreement with the network provider (which provided a toll free SMS service) after April 2011. This challenge brought to light the influence providers can have on the success or failure of an SMS-based project. Following the suspension, CHWs were unable to work with the system for a few consecutive weeks; and based on data from interviews, many shared that they were receiving backlogged messages, that is, appointment reminders for women who had already delivered. Furthermore, CHWs were unable to register newly pregnant women to the system, and returned to using Pregnancy Tracking forms. Most CHWs resorted to using their own phone credit to send text messages to the system and were reimbursed by the MVP office for this expense. Future SMS-projects piloted, either in MVP program sites or by any group or government considering mHealth interventions, will need to ensure that agreements with network providers are secure, based on long-term and mutually beneficial relationships.

Our system was easily set-up on the already existing ChildCount + platform rolled out across all MVP sites, including Sauri, Kenya. A small initial investment in equipment and programmer time was sufficient to set up the SMS-based alert system, and running costs during the project were minimal. Other programs interested in setting a similar reminder system may not have access to the CC+ backbone that has simplified registration of patients in our case. This should not however detract other groups from exploring similar SMS-based tracking systems. A complete standalone system that does not rely on CC+ can be devised, and our study only serves to demonstrate the impact of the tool following its implementation.

Based on results found, one improvement to the system would be to gradually complement the CHWs component within the workflow by having text messages sent directly to women using their mobile phone number as the unique identifier. Currently, as described previously, CHWs are a key component of the system, and its effectiveness is reliant on their performance. This option will remove the system’s complete dependence on CHWs, and pregnant women will be included in the Closed User Group (CUG) and contract agreement with the network provider. A challenge with this option is that not all women in households in Sub-Saharan Africa have access to a phone-only 20% of women interviewed mentioned that they owned a phone. Most phones in the households belong to the husband or partner, which would raise issues of confidentiality. Further, there will be additional costs to the project should women be included in the CUG. Hence this option will be feasible should mobile phone penetration rates increase and more women opt-in into the idea of having reminders sent to their phones.

## Conclusions

In the studied sample, women enrolled in the APAS were more likely to undergo the 4 recommended ANC visits and were more likely to make the recommended 6 baby follow-ups compared to women not enrolled in the APAS. Registration in the APAS also eliminated vertical HIV transmission rates in this sample. Our study shows that the use of a combination of CHW programs and mHealth tools not only strengthens adherence to ANC and PNC, but may also allow communities well integrated into the primary health system to reach closer to the goal of elimination of vertical HIV transmission in PMTCT programs.

## Endnotes

^a^It is important to note that at no time is the patient’s HIV status revealed to the CHW, this program follows all pregnant women and children under 18 months as to not de facto reveal status.

^b^There were 109 CHWs in the MVP cluster during the time of the study.

^c^Babies are tracked in the CC+ system up to 18 months. Interviews were conducted with new mothers if their babies were aged 18 months or less.

^d^Statistics collected from Ramula Health Center.

## Additional files

Additional file 1:
**ChildCount+ Form FP: Pregnancy Demographics.** Upon presentation to the clinic for the 1^st^ ANC visit, each woman has her assigned CHW fill out Form FP and the CHW sends the form to the APAS at the end of the day. Additional file 2:
**ChildCount+ Form FP2: Initial Antenatal Visit.** After initial screening by the CHW, each pregnant woman visits the nurse who fills out Form FP2, which is automatically sent to the APAS at the end of the day. Additional file 3:
**ChildCount+ Form FP3: Follow Up Visit Log.** Each CHW completes form FP3 for every follow up visit of each pregnant woman in his or her household catchment area, and submits the form to the APAS at the end of the day. Additional file 4:
**Frequency of population characteristic by ANC visits for women presenting for 1** ^**st**^ **ANC visit in 2** ^**nd**^ **trimester of pregnancy.** Each characteristic shows differences in frequency per sub-category. Additional file 5:
**Frequency of population characteristic by baby follow-ups for all women.** Each characteristic shows differences in frequency per sub-category. Additional file 6:
**Frequency of population characteristics by baby’s HIV status at 18 months for HIV positive women.** Each characteristic shows differences in frequency per sub-category.

## Abbreviations

ANC: Antenatal Care
APAS: ANC and PMTCT Adherence System
ART: Antiretroviral Therapy
CC+: ChildCount Plus
CHW: Community Health Worker
CUG: Closed User Group
HEI: HIV-Exposed Infant
HIV: Human Immunodeficiency Virus
ID: Identification
MCH: Maternal and Child Health
mHealth: Mobile Health
MVP: Millennium Villages Project
PCR: Polymerase Chain Reaction
PMTCT: Prevention of Mother-to-Child Transmission of HIV
SMS: Short Message System
TB: Tuberculosis
