# Supplementary material for: Evaluation of the impact of a mobile health system on adherence to antenatal and postnatal care and prevention of mother-to-child transmission of HIV programs in Kenya
Source: BMC Public Health. 2015 Feb 7;15:102. doi: 10.1186/s12889-015-1358-5 (PMC4328364; doi:10.1186/s12889-015-1358-5)
Supplement: Additional file 1: — ChildCount+ Form FP: Pregnancy Demographics. Upon presentation to the clinic for the 1st ANC visit, each woman has her assigned CHW fill out Form FP and the CHW sends the form to the APAS at the end of the day. [file 12889_2015_1358_MOESM1_ESM.pdf]

# ChildCount+ Form FP:PREGNANCY DEMOGRAPHICS

Facility Name: ______________________

**Focus on: Pregnant Women**

PREGNANCY DEMOGRAPHICS: For pregnant women with a HEALTH ID that need to have a pregnancy recorded in the CHW data system. **This form only needs to be filled out one time per woman per pregnancy.**

| Date | PREGNANT WOMAN'S HEALTH ID | Pregnancy Demographics | Married (Y-N-U) [HEALTH ID if available] | Number of Pregnancies | Number of children from that mother living in the home with her currently |
|---|---|---|---|---|---|
| | | +PD | | | |
| | | +PD | | | |
| | | +PD | | | |
| | | +PD | | | |
| | | +PD | | | |
| | | +PD | | | |
| | | +PD | | | |
| | | +PD | | | |
| | | +PD | | | |
| | | +PD | | | |
| | | +PD | | | |
| | | +PD | | | |
| | | +PD | | | |
| | | +PD | | | |
| | | +PD | | | |
| | | +PD | | | |
| | | +PD | | | |
| | | +PD | | | |
| | | +PD | | | |
| | | +PD | | | |
| | | +PD | | | |
| | | +PD | | | |
| | | +PD | | | |
| | | +PD | | | |
| | | +PD | | | |
| | | +PD | | | |
| | | +PD | | | |
| | | +PD | | | |
| | | +PD | | | |
| | | +PD | | | |
| | | +PD | | | |
| | | +PD | | | |

| Date | PREGNANT WOMAN'S HEALTH ID | Pregnancy Demographics | Married (Y-N-U) [HEALTH ID if available] | Number of Pregnancies | Number of children from that mother living in the home with her currently |
|---|---|---|---|---|---|
| | | +PD | | | |
| | | +PD | | | |
| | | +PD | | | |
| | | +PD | | | |
| | | +PD | | | |
| | | +PD | | | |
| | | +PD | | | |
| | | +PD | | | |
| | | +PD | | | |
| | | +PD | | | |
| | | +PD | | | |
| | | +PD | | | |
| | | +PD | | | |
| | | +PD | | | |
| | | +PD | | | |
| | | +PD | | | |
| | | +PD | | | |
| | | +PD | | | |
| | | +PD | | | |
| | | +PD | | | |
| | | +PD | | | |
| | | +PD | | | |
| | | +PD | | | |
| | | +PD | | | |
| | | +PD | | | |
| | | +PD | | | |
| | | +PD | | | |
| | | +PD | | | |
| | | +PD | | | |
| | | +PD | | | |
| | | +PD | | | |
| | | +PD | | | |
