# Supplementary material for: Evaluation of the impact of a mobile health system on adherence to antenatal and postnatal care and prevention of mother-to-child transmission of HIV programs in Kenya
Source: BMC Public Health. 2015 Feb 7;15:102. doi: 10.1186/s12889-015-1358-5 (PMC4328364; doi:10.1186/s12889-015-1358-5)
Supplement: Additional file 2: — ChildCount+ Form FP2: Initial Antenatal Visit. After initial screening by the CHW, each pregnant woman visits the nurse who fills out Form FP2, which is automatically sent to the APAS at the end of the day. [file 12889_2015_1358_MOESM2_ESM.pdf]

ChildCount+ Form FP2: Initial Antenatal Visit

Facility Name ____

PREGNANCY ENROLLMENT: For health centers to enroll pregnant women at first visit

Date	PREGNANT WOMAN'S CHILDCOUNT+ HEALTH ID	Initial Antenatal Visit	Expected Date of Delivery [DDMMYY]	HIV Test	HIV+ (Y-N-U- NC)	Blood Drawn for CD4 [Optional] (Y-N-U)	Next Appointment	Date of Next Appointment [DDMMYY]
		+PF		+HT			+AP	
		+PF		+HT			+AP	
		+PF		+HT			+AP	
		+PF		+HT			+AP	
		+PF		+HT			+AP	
		+PF		+HT			+AP	
		+PF		+HT			+AP	
		+PF		+HT			+AP	
		+PF		+HT			+AP	
		+PF		+HT			+AP	
		+PF		+HT			+AP	
		+PF		+HT			+AP	
		+PF		+HT			+AP	
		+PF		+HT			+AP	
		+PF		+HT			+AP	
		+PF		+HT			+AP	
		+PF		+HT			+AP	
		+PF		+HT			+AP	
		+PF		+HT			+AP	
		+PF		+HT			+AP	
		+PF		+HT			+AP	
		+PF		+HT			+AP	
		+PF		+HT			+AP	
		+PF		+HT			+AP	
		+PF		+HT			+AP	
		+PF		+HT			+AP	
		+PF		+HT			+AP	
		+PF		+HT			+AP	
		+PF		+HT			+AP	
		+PF		+HT			+AP	
