# Supplementary material for: Evaluation of the impact of a mobile health system on adherence to antenatal and postnatal care and prevention of mother-to-child transmission of HIV programs in Kenya
Source: BMC Public Health. 2015 Feb 7;15:102. doi: 10.1186/s12889-015-1358-5 (PMC4328364; doi:10.1186/s12889-015-1358-5)
Supplement: Additional file 3: — ChildCount+ Form FP3: Follow Up Visit Log. Each CHW completes form FP3 for every follow up visit of each pregnant woman in his or her household catchment area, and submits the form to the APAS at the end of the day. [file 12889_2015_1358_MOESM3_ESM.pdf]

ChildCount+ Form FP3: Follow Up Visit Log

Facility Name ____

For health centers to record follow up antenatal and 18 month and under follow-ups
If the child has reached **18 months, please enter 000000 to indicate no next visit**

Date	Health ID	Next Appointment	Date of Next Appointment [DDMMYY]
		+AP	
		+AP	
		+AP	
		+AP	
		+AP	
		+AP	
		+AP	
		+AP	
		+AP	
		+AP	
		+AP	
		+AP	
		+AP	
		+AP	
		+AP	
		+AP	
		+AP	
		+AP	
		+AP	
		+AP	
		+AP	

Date	Health ID	Next Appointment	Date of Next Appointment [DDMMYY]
		+AP	
		+AP	
		+AP	
		+AP	
		+AP	
		+AP	
		+AP	
		+AP	
		+AP	
		+AP	
		+AP	
		+AP	
		+AP	
		+AP	
		+AP	
		+AP	
		+AP	
		+AP	
		+AP	
		+AP	
		+AP	

Date	Health ID	Next Appointment	Date of Next Appointment [DDMMYY]
		+AP	
		+AP	
		+AP	
		+AP	
		+AP	
		+AP	
		+AP	
		+AP	
		+AP	
		+AP	
		+AP	
		+AP	
		+AP	
		+AP	
		+AP	
		+AP	
		+AP	
		+AP	
		+AP	
		+AP	
		+AP	
