# Supplementary figures and images for: Evaluation of the impact of a mobile health system on adherence to antenatal and postnatal care and prevention of mother-to-child transmission of HIV programs in Kenya
Source: BMC Public Health. 2015 Feb 7;15:102. doi: 10.1186/s12889-015-1358-5 (PMC4328364; doi:10.1186/s12889-015-1358-5)

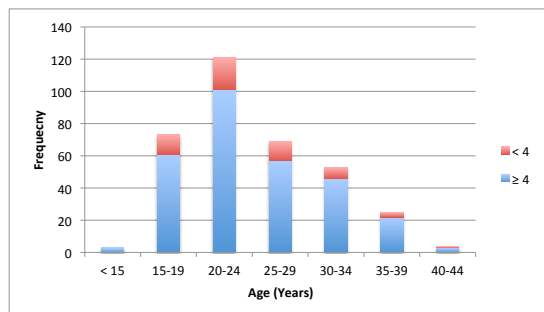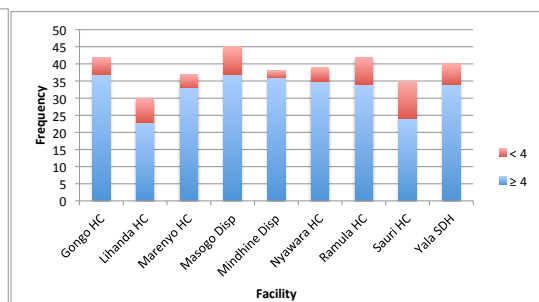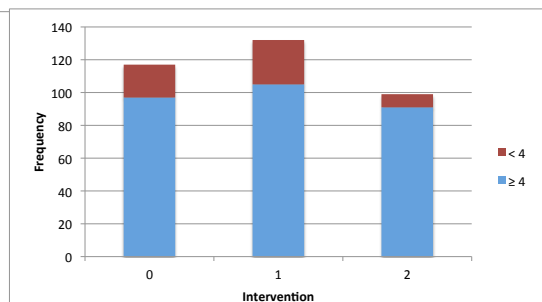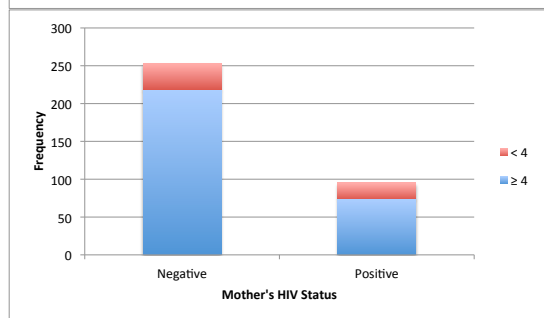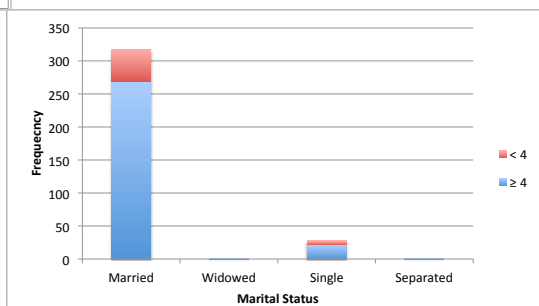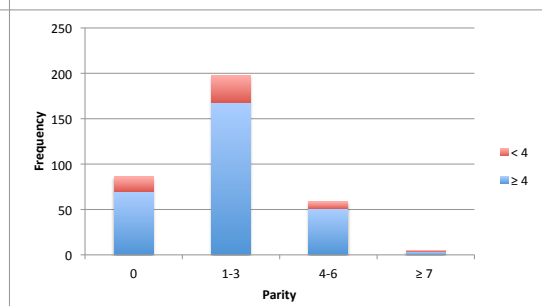

Supplement: Additional file 4: — Frequency of population characteristic by ANC visits for women presenting for 1 st ANC visit in 2 nd trimester of pregnancy. Each characteristic shows differences in frequency per sub-category. [file 12889_2015_1358_MOESM4_ESM.pdf]

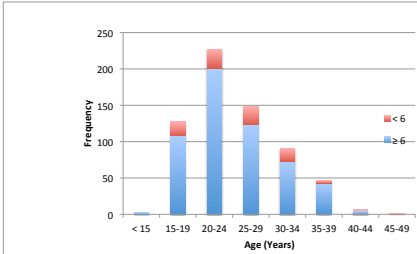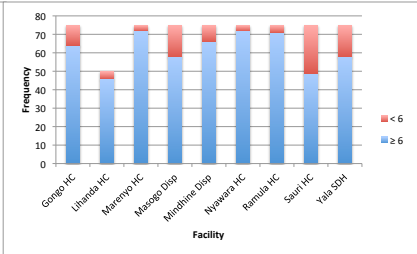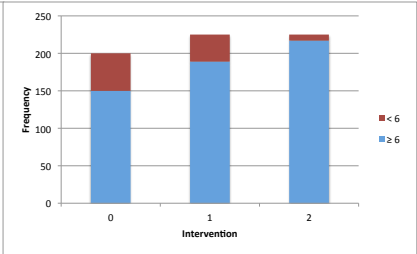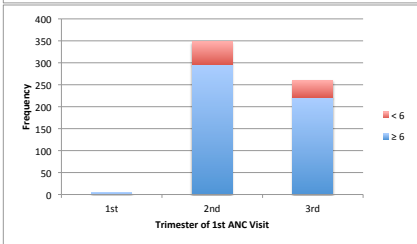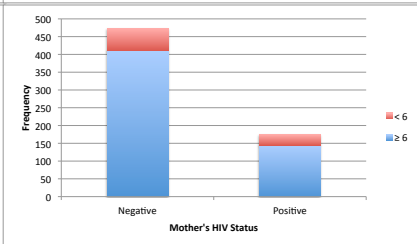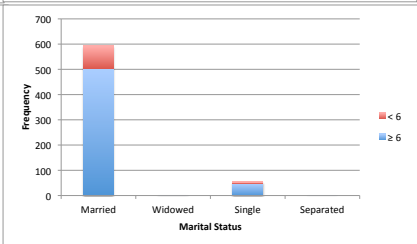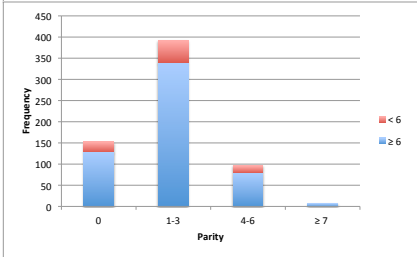

Supplement: Additional file 5: — Frequency of population characteristic by baby follow-ups for all women. Each characteristic shows differences in frequency per sub-category. [file 12889_2015_1358_MOESM5_ESM.pdf]

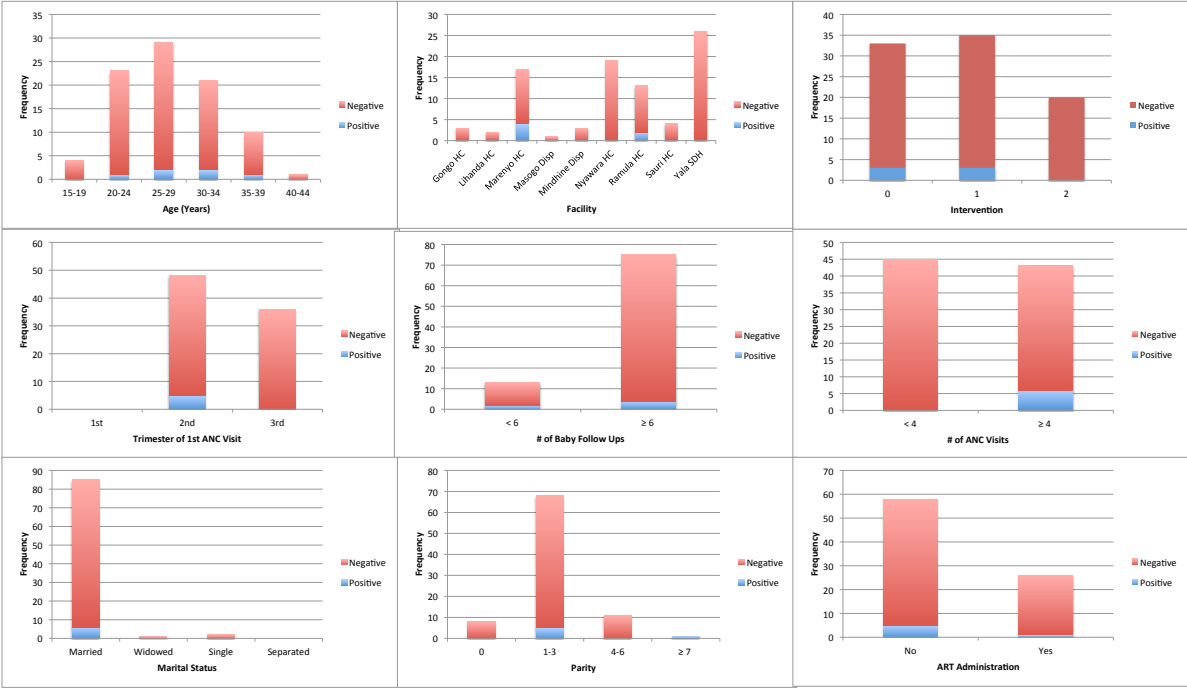

Supplement: Additional file 6: — Frequency of population characteristics by baby’s HIV status at 18 months for HIV positive women. Each characteristic shows differences in frequency per sub-category. [file 12889_2015_1358_MOESM6_ESM.pdf]
